# Effects of exogenous selenium application on nutritional quality and metabolomic characteristics of mung bean (*Vigna radiata* L.)

**DOI:** 10.3389/fpls.2022.961447

**Published:** 2022-08-18

**Authors:** Kexin Wang, Yuhao Yuan, Xinyu Luo, Zhaoyang Shen, Yinghui Huang, Haolu Zhou, Xiaoli Gao

**Affiliations:** State Key Laboratory of Crop Stress Biology in Arid Areas, College of Agronomy, Northwest A&F University, Yangling, Shaanxi, China

**Keywords:** selenium biofortification, metabolome, nutrition, biomarkers, quality

## Abstract

Selenium (Se) biofortification is an important strategy for reducing hidden hunger by increasing the nutritional quality of crops. However, there is limited metabolomic information on the nutritional quality of Se-enriched mung beans. In this study, physiological assays and LC–MS/MS based widely targeted metabolomics approach was employed to reveal the Se biofortification potential of mung bean by evaluating the effect of Se on mung bean nutraceutical compounds and their qualitative parameters. Physiological data showed that foliar application of 30 g ha^−1^ Se at key growth stages significantly increased the content of Se, protein, fat, total phenols, and total flavonoids content in two mung bean varieties. Widely targeted metabolomics identified 1,080 metabolites, among which L-Alanyl-L-leucine, 9,10-Dihydroxy-12,13-epoxyoctadecanoic acid, and 1-caffeoylquinic acid could serve as biomarkers for identifying highly nutritious mung bean varieties. Pathway enrichment analysis revealed that the metabolic pathways of different metabolites were different in the Se-enriched mung bean. Specifically, P1 was mainly enriched in the linoleic acid metabolic pathway, while P2 was mainly enriched in the phosphonate and phosphinate metabolic pathways. Overall, these results revealed the specific Se enrichment mechanism of different mung bean varieties. This study provides new insights into the comprehensive improvement of the nutritional quality of mung beans.

## Introduction

The distribution of selenium (Se) in the soil is influenced by climate–soil interactions (Alcântara et al., 2022). Low-Se soils are most likely to occur in arid environments and areas with high pH and low clay content. Conversely, areas with low to moderate precipitation but relatively low aridity and high clay content are likely to have higher soil Se concentrations. At present, the average content of Se in the soil in the world is 0.4 mg kg^−1^. However, due to climate change and changes in soil organic carbon content, global Se content will be reduced to 0.316 mg kg^−1^ by the end of the 21st century, and farmland will lose more soil Se (Jones et al., 2017). Se deficiency affects about 500 million to 1 billion people worldwide (Wang et al., 2013a). According to FAO/WHO, the dietary Se supply should be 55–70 μg/day, with a suitable range of 50–250 μg/day, and a maximum dose of 400 μg/day (Kieliszek and Błażejak, 2013). However, the daily intake of Se among the global human population is far below the recommended standard (Strand et al., 2018). At present, it is not clear whether Se is an essential element for plant growth and development. However, Se fertilization can alleviate plant salt stress, reduce the accumulation of cadmium, lead and arsenic in plant tissues, improve plant growth, physiological and biochemical parameters, and improve stress resistance (Hu et al., 2014; Rasool et al., 2020, 2022; Schiavon et al., 2020; Sun et al., 2021). For human health, Se plays anti-oxidative, anti-viral, and anti-cancerous role. It is also used to treat Keshan disease, hormone imbalance, and Kashin–Beck disease (Tapiero et al., 2003; Nacamulli et al., 2010; Liu et al., 2018; Zhou et al., 2018). However, severe excessive selenium supply affects the digestive system, nervous system, respiratory system, circulatory system, and skin epidermis, resulting in serious health issues (Steinbrenner and Sies, 2013; Kieliszek, 2019; Gać et al., 2021). Therefore, considering the possible side effects resulting from excessive Se supply, we should be cautious about supplementation. Gissel-Nielsen (1986) revealed that foliar application of Se increases primary Se content in crops. Wang et al. (2013b) optimized Se biofortification in rice to improve the enrichment efficiency of Se in the grains and enhance the antioxidant properties of rice. Notably, most studies on Se-enriched crops have mainly focused on improving agronomic biofortification to prevent Se deficiency. However, research on improving the nutrition of food crops through Se biofortification strategies has lagged. Therefore, there is a need to explore the key functional substances in Se-enriched crops. This will be instrumental in preventing and treating human diseases to improve human health globally.

Mung bean [*Vigna radiata* (L.) *Wilczek*] is one of the most important edible legume crops globally. Currently, mung bean is planted in more than 6 million hectares (about 8.5% of the global legume area) worldwide, mainly concentrated in China, India, Pakistan, Myanmar, Canada, and the United States, with the highest consumption in Asian countries (Dahiya et al., 2015; Hou et al., 2019). Mung beans are high in protein, moderately starchy, low in fat, and contain various biologically active substances, including flavonoids (such as quercetin-3-glycoside, quercetin, myricetin, kaempferol) and phenolic acids (such as p-hydroxy benzene; Tang et al., 2014b; Chai et al., 2018). Compared with other beans, mung beans contain more digestible proteins and produce less phytic acid. Mung bean is also considered to be an ideal healthy food for human beings (Nair et al., 2013). Studies have shown that the Se biofortification potential of legumes is stronger than that of other plant categories such as Gramineae, Compositae, and Umbelliferae. Se fertilization can significantly promote the growth of legumes and increase Se accumulation (Djanaguiraman et al., 2010; Quinn et al., 2011). Se and sulfur have very similar properties. Specifically, the metabolic pathway of Se is similar to that of sulfur (Schiavon et al., 2015). Se binds to proteins to form seleno-proteins, which act as powerful antioxidants in plant metabolism. Particularly, seleno-proteins enhance the scavenging of reactive oxygen species (ROS) by enzymatic (SOD, CAT, and APX) and non-enzymatic (ascorbic acid, flavonoids, and tocopherols) compounds to protect plant tissue from oxidative stress (Takata et al., 2021; Alcântara et al., 2022). In addition, studies have found that plants convert Se to seleno-methionine (Se-Met), which is incorporated into proteins in place of methionine (Met). Se-Met accounts for more than 50% of the total Se content of plants. Therefore, when plants are biofortified with low concentrations of Se, higher levels of protein are required to combine with Se to form seleno-proteins and seleno-amino acids that are beneficial to human health. Mung beans contain high levels of proteins and amino acids, which significantly contributes to their nutritional content (Hadidi et al., 2021). Specifically, mung bean kernels contain about 20.97%–31.32% protein, which is higher than soybeans (1%8–22%) and kidney beans (20%–30%), and twice the protein content of corn (6%–12%; Anwar et al., 2007; Shevkani et al., 2015; Xu et al., 2015; Ai and Jane, 2016; Hadidi et al., 2021). Thus, mung bean has a greater potential for Se enrichment than other crops.

Metabolomics applies high-throughput chemical analysis techniques to analyze small molecule metabolites qualitatively and quantitatively in biological samples. In recent years, metabolomic analysis has been applied to a large number of biological studies ranging from plant physiological metabolism to the development of personal metabolomics, contributing to human understanding of complex molecular interactions within biological systems (Bino et al., 2004). Currently, the two main analytical technologies used in metabolomics are nuclear magnetic resonance (NMR) and mass spectrometry (MS; Lu et al., 2017; Li et al., 2021). NMR-based metabolic profiling is a quick, expedient, and efficient technology for the screening and identification of similar biological samples. It is non-destructive, selective, and very proficient at mapping metabolic pathways. Moreover, its high reproducibility makes it a powerful tool in plant metabolomics research (Razzaq et al., 2019). MS analysis technology is characterized by high sensitivity, high speed, and high throughput, which can detect extremely low concentrations of metabolites with higher resolution and reliability of dynamic range (Li et al., 2021). While MS analytical techniques account for more than 80% of published metabolomics studies to date, there is still considerable interest in using NMR-based methods for metabolomic studies. The number of NMR-based metabolomics/metabonomics papers has steadily increased over the past 15 years (Emwas et al., 2019). Moreover, the combination of LC–MS and NMR detection methods further increases the diversity of detection methods and aids in the identification of primary and secondary metabolites (Bingol et al., 2015; Boiteau et al., 2018). Metabolomics profiling methods can be targeted or untargeted depending on the detection target. Targeted metabolomic analysis is sensitive and accurate, but metabolite detection is limited, and it is difficult to meet research needs when complex metabolic pathways are involved. Untargeted metabolomic assays can be used to detect hundreds or thousands of metabolites simultaneously, but they have low sensitivity and relatively poor quantitative accuracy. Widely targeted metabolomics combines the advantages of both untargeted and targeted metabolite detection methods, it can simultaneously quantify hundreds of known metabolites and nearly 1,000 known and unknown metabolites by using the innate Q TRAP mass spectrometry in multiple reaction monitoring (MRM) mode (Sawada et al., 2009; Chen et al., 2013; Yang et al., 2019). Previously, scholars applied metabolomics techniques to study mung bean, for example, Tang et al. (2014a) used metabolomics technology to study the changes of polyphenol metabolites in mung bean seeds and their germination process, identified the biomarkers in the germination process of mung bean, and discussed the main metabolic and transformation pathways of flavonoids and phenolic acids in the germination process. Chen et al. (2019) used NMR spectroscopy to study the metabolomic analysis of energy-regulated germination and sprouting of mung beans. The results indicate that adequate energy supply is essential for the nutritional quality and metabolism of mung bean sprouts. Lyu et al. (2022) used widely targeted metabolomics to analysis characterizes the phenolic compounds profiles in mung bean sprouts under sucrose treatment and found that sucrose induced significant changes of different phenolic compounds. Na Jom et al. (2011) used metabolomics to analyze the changes of metabolites during germination of mung bean and found that with the prolongation of germination time, the contents of monosaccharides, organic acids, and amino acids in mung bean sprouts increased significantly, while the content of fatty acid methyl esters decreased. However, to the best of our knowledge, the feasibility of Se biofortification in mung beans and its impact on the nutritional value of mung beans have not been evaluated. Thus, this study aimed to evaluate the Se biofortification potential of mung beans and examine the effect of Se supplementation on protein, fat, phenolic compounds content, and qualitative parameters of metabolites in mung beans.

## Materials and methods

### Plant materials and treatments

Mung bean varieties Baolv200520-2 (P1) and Lvfeng2 (P2) were used in this study. Baolv200520-2 is black-skinned mung bean, whereas Lvfeng2 is green-skinned. A field experiment was conducted in 2021 at Northwest Agriculture and Forestry University [Yangling, Shaanxi, China (34°16′56′N; 108°4′28′E)]. The total Se content of the soil in this area was 0.173 mg kg^−1^, which belongs to the low-Se area [the soil’s total Se content of 0.1–0.6 mg kg^−1^ was Se-deficient soil (Gupta and Gupta, 2000)]. Therefore, it was valuable to conduct research on Se-enriched mung bean in this area. Na_2_SeO_3_ (Sigma-Aldrich, the United States) was used as the Se source. Four treatments with Se content of 0 (CK), 15 (Y1), 30 (Y2), and 45 (Y3) g ha^−1^ were set up. They were prepared as 0, 15, 30, and 45 mg L^−1^ Se solutions. Each experimental plot area is 30 m^2^, and each treatment uses 6 plots. Different concentrations of Se solution was sprayed on the plants once at the beginning of the flowering stage. To avoid water evaporation, after 16: 00, we sprayed the front and back of the whole leaf evenly with a sprayer, until all the leaves were soaked and dripping. The Se solution was replaced with distilled water as a control (CK), which was replenished within 6 h in case of rain, and the different plots were separated by plastic film during spraying. At maturity, 30 mung bean plants from each treatment were randomly collected from the experimental site. Ten plants were considered as one biological sample, and each treatment had three biological samples. After plant collection, the pods were removed and stored at –80°C refrigerator until further analysis.

### Determination of Se, protein, fat, total phenolic and phytic acid contents

The mung bean samples were dried in a freeze dryer, ground, and passed through an 80-mesh sieve. The Se content of the samples was determined as described by Wang et al. (2020). The protein content was determined using the Kjeldahl method and calculated according to the nitrogen content (N × 6.25; Gao, 2006). The fat content was determined according to the Soxhlet extraction method using petroleum ether (boiling point 60°C–90°C) as the solvent (Gao, 2006). Total phenolic and total flavonoids content in the samples was assessed based on the method described by Xu and Chang (2008). The phytic acid content of the samples was determined as described by Davies and Reid (1979).

### Sample preparation and metabolite extraction

The preparation of mung bean samples and the extraction of metabolites were performed at MetWare Biological Science and Technology Co., Ltd. (Wuhan, China). All chemical reagents used were of analytical grade. Methanol and acetonitrile were purchased from Merck (Germany), while standard reagents (dimethyl sulfoxide) were purchased from BioBioPha and Sigma-Aldrich Co., Ltd. (the United States). The samples were dried using a freeze dryer (Scientz-100F, Shanghai, China) and then pulverized using a mixer with zirconia beads (MM 400, Retsch, Shanghai, China) at 30 Hz for 1.5 min. Subsequently, 100 mg of the lyophilized powder was weighed and dissolved in 1.2 ml of 70% methanol solution, spun six times at 30 s intervals, and kept at 4°C in a refrigerator overnight. The next day, the samples were centrifuged at 16,000 *g* for 10 min, and the supernatant was filtered through a microporous membrane filter (SCAA-104, 0.22 μm pore size; ANPEL, Shanghai, China) and then subjected to UPLC–MS/MS analysis.

### ESI-Q TRAP-MS/MS analysis

Sample extracts were analyzed using the UPLC-ESI-MS/MS system (UPLC, SHIMADZU Nexera X2; MS, Applied Biosystems 4,500 Q TRAP). Analytical conditions used were as follows: UPLC: Column: Agilent SB-C18 1.8 μm, 2.1 mm × 100 mm; mobile phase: solvent A was ultrapure water (added with 0.1% formic acid), solvent B was acetonitrile (added with 0.1% formic acid); elution gradient: 0.00 min, the ratio of solvent B was 5%, and the ratio of solvent B increased linearly within 9 min to 95% and remained at 95% for 1 min, 10.00–11.10 min, the proportion of solvent B was reduced to 5%, and equilibrated at 5% for 14 min; flow rate was 0.35 ml/min; column temperature was 40°C; injection volume was 4 μl. The effluent was alternatively connected to an ESI-triple quadrupole linear ion trap (Q TRAP)-MS.

Linear ion trap (LIT) and triple quadrupole (QQQ) scans were acquired on a triple quadrupole linear ion trap mass spectrometer (Q TRAP) AB4500 Q TRAP UPLC/MS/MS system, equipped with an ESI Turbo ion spray interface that can be analyst 1.6.3 software (AB Sciex) controlled the operation of positive and negative ion modes. ESI source operation parameters were as follows: an ion source, temperature of 550°C; ion spray voltage (IS) of 5,500 V/−4,500 V; ion source gas I (GSI), gas II (GSII). The settings were 50 psi and 60 psi, the curtain air (CUR) was 25.0 psi, and the collision-induced ionization (CAD) parameter was set to high. Instrument tuning and mass calibration were performed with 10 and 100 μmol/L polypropylene glycol solutions in QQQ and LIT modes, respectively. The collision gas (nitrogen) was set to wait for the MRM experiment to obtain the QQQ scan results. Each ion pair was scanned in triple quadrupoles based on declustering potential (DP) and collision energy (CE). Based on the eluted metabolites, a specific set of MRM transitions was monitored in each period.

### Data analysis

All data for quantitative analysis are presented as mean ± standard deviation (SD). One-way analysis of variance (ANOVA) was used to determine the difference among various treatments at *p* < 0.05. Metabolic data were log_2_ transformed before statistical analysis to ensure normality and normalization. Principal component analysis (PCA), cluster analysis (HCA), and Venn diagram of metabolites were performed on the metabolites of 12 mung bean samples using Rv3.5.0 software. According to Orthogonal Partial Least Squares Discriminant Analysis (OPLS-DA) prediction model stability reliability, fold change, and VIP value were used to further screen differential metabolites to study the specific accumulation of metabolites. The Kyoto Encyclopedia of Genes and Genomes (KEGG) database was used to annotate metabolites in Se-enriched mung beans of different varieties at *p* < 0.05. Graphs were drawn using GraphPad Prism v6.01.

## Results

### Effect of Se application on Se and nutrient contents in mung bean

**Figure 1:**
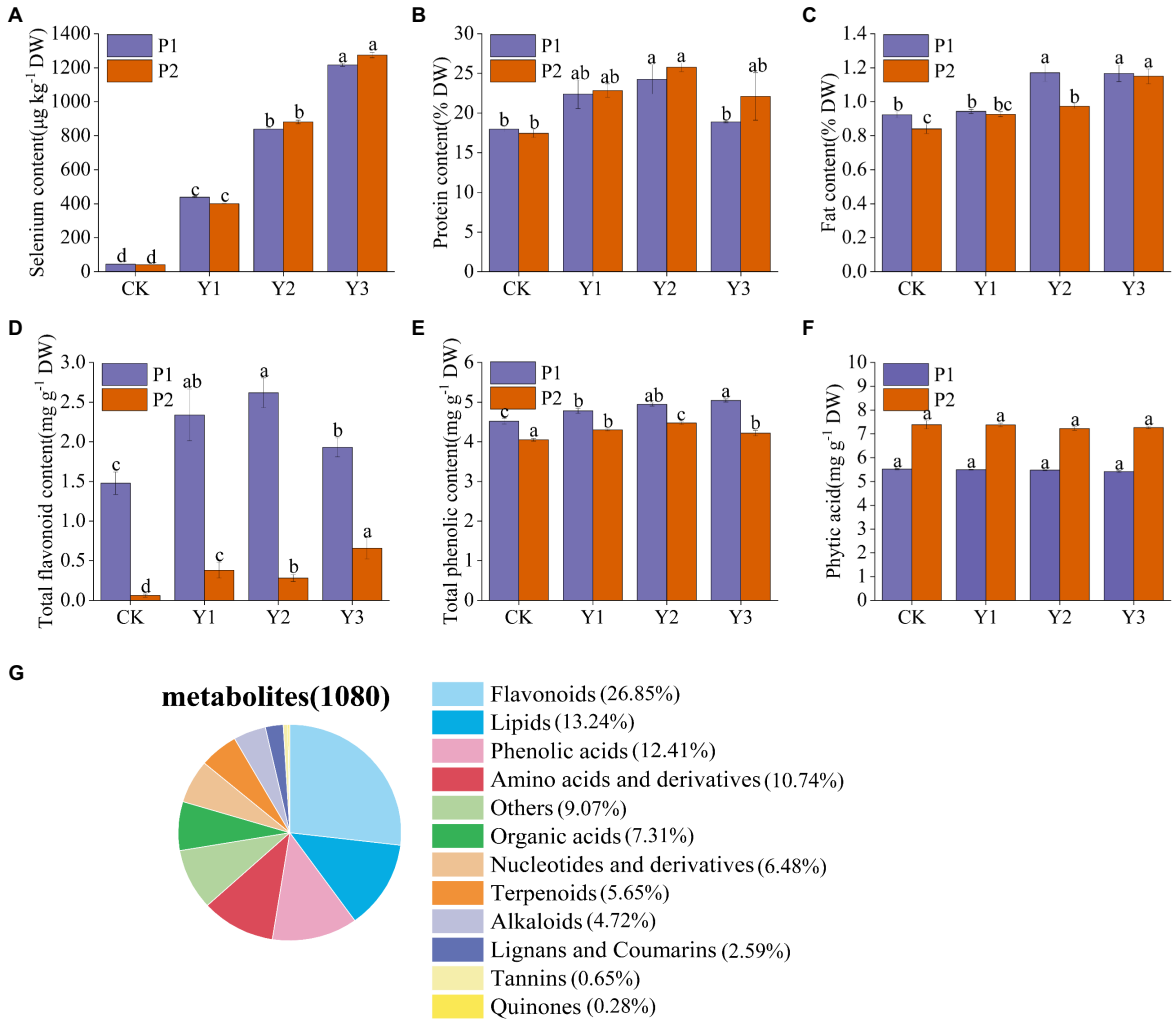
Effects of Se fertilization on Se content of mung beans **(A)**, protein **(B)**, fat **(C)**, total flavonoids **(D)**, total phenols **(E)**, phytic acid **(F)**, and classification of 1,080 metabolites identified in mung bean samples **(G)**. Data represent mean values ± SD of three independent measurements. Different letters indicate significantly different values (*p* < 0.05).

Se content of the two mung bean varieties, P1 and P2, increased with the increase in Se concentration. At the concentration of Y1, the Se contents of P1 and P2 were 438.4 ± 4.2 μg kg^−1^ and 399.6 ± 2.6 μg kg^−1^, respectively. At Y2, the Se contents of P1 and P2 were 839.6 ± 7.5 μg kg^−1^ and 881.4 ± 10.6 μg kg^−1^, while at Y3, the Se contents were 1216.6 ± 8.6 μg kg^−1^ and 1274.5 ± 16.3 μg kg^−1^, respectively (Figure 1A). Due to the narrow range between the optimal and toxic Se concentrations, we considered nutritional indicators (protein, fat), bioactive substances (total flavonoids, total phenols), and antinutritional factor (phytic acid) in selecting the optimal Se concentration. The contents of protein, fat and total flavonoids in P1 were highest at Y2 and slightly decreased at Y3. In P2, protein and total phenols were highest at Y2 and slightly decreased at Y3 (Figures 1B–E). However, Se application did not affect the phytic acid content of mung bean (Figure 1F). Therefore, by considering nutrients and bioactive substances, Y2 treatment was selected as the most beneficial to the absorption of Se by mung beans, promoting the growth and enriching the nutrient content of crops. Thus, Y2 treatment was used in subsequent experiments.

### Metabolic profiling of Se-enriched mung bean

Extensive targeted metabolomics technology under the LC–MS/MS platform was employed to elucidate the changes of metabolites in the two Se-enriched mung bean varieties. A total of 1,080 metabolites were detected from 12 biological samples. These includes 12 primary metabolites, such as flavonoids (26.85%), lipids (13.24%), phenolic acids (12.41%), amino acids and their derivatives (10.74%), other (9.07%), organic acids (7.31%), nucleotides and derivatives (6.48%), terpenoids (5.65%), alkaloids (4.72%), lignans and coumarins (2.59%), tannins (0.65%), and quinones (0.28%; Figure 1G; Supplementary Table S1). Notably, metabolites related to nutrients (flavonoids, lipids, phenolic acids, amino acids, and derivatives) accounted for 63.24% of the total metabolites.

### Multivariate statistical analysis reveals differences between metabolic profiles of different Se-enriched mung bean varieties

**Figure 2:**
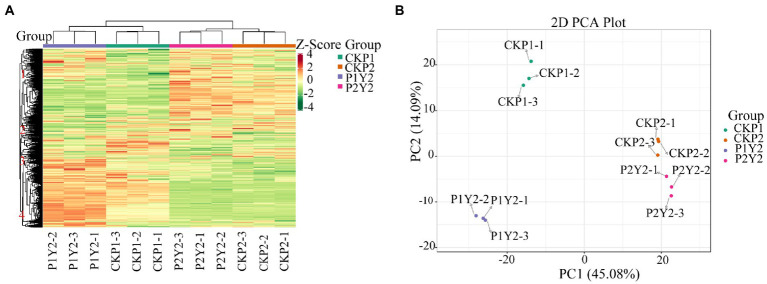
Hierarchical cluster analysis (HAC) **(A)**, red indicates high abundance, and purple indicates low abundance. Principal component analysis (PCA) **(B)**.

The results of cluster analysis (HCA) showing the accumulation patterns of metabolites in CKP1, P1Y2, CKP2, and P2Y2 treatment groups are shown in Figure 2A. The results showed that there were significant differences in metabolite accumulation between the different groups. The metabolites were divided into four clusters. Metabolites in clusters 1 and 2 were most abundant in the CKP2 and P2Y2 groups, while those in clusters 3 and 4 were most abundant in the P1Y2 group. The CK1 group was intermediate, and the CKP2 and P2Y2 groups were the lowest. These results indicate that the metabolites of Se-enriched mung bean differed significantly between the two varieties. Notably, different biological replicates clustered together, indicating good homogeneity among biological replicates and high reliability of the data.

The PCA results showed that the metabolites were separated between different treatments (Figure 2B), indicating that the metabolite contents varied significantly among the treatment groups. The first principal component (PC1) separated the two varieties, and its contribution value reached 45.08%. CKP2 and P2Y2 were closely clustered, while CKP1 and P1Y2 were separated. The second principal component (PC2) explained 14.09% of the properties of the original dataset, indicating variations between different Se-enriched treatments. The metabolite changes caused by genotypic differences were larger than those caused by Se-enriched treatment, suggesting that genotypic influence was the main source of metabolite differences in samples. Of note, different varieties had different sensitivity to Se treatment.

### Differential metabolite analysis

**Figure 3:**
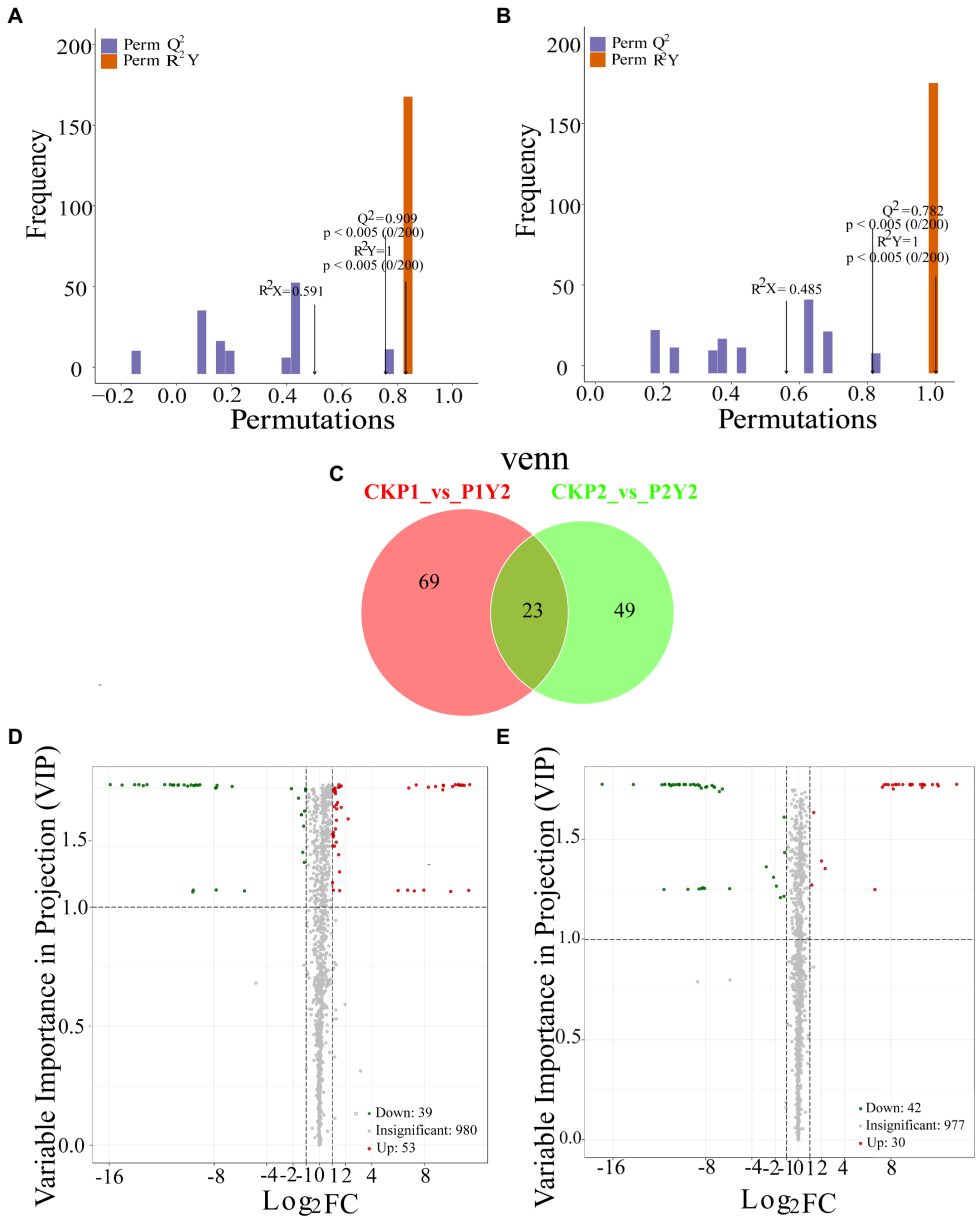
Differential analysis of metabolites in Se-enriched mung beans. OPLS-DA model plots of Se-enriched mung bean **(A,B)**. Venn diagrams showing the overlapping and specific differential metabolites **(C)**. Volcano plots showing the differences in the expression levels of metabolites in Se-enriched mung beans, red spots indicate upregulated differentially expressed metabolites; green spots indicate downregulated differentially expressed metabolites, and grey spots indicate detected metabolites, the differences were not significant at *p* < 0.05 **(D,E)**.

OPLS-DA analysis is a multivariate statistical analysis method with supervised pattern recognition (Figures 3A,B), which can effectively eliminate irrelevant effects while screening differential metabolites. Pairwise analysis of CKP1, P1Y2, CKP2, and P2Y2 was performed using OPLS-DA to plot the scores. In this model, R^2^X and R^2^Y represent the interpretation rate of the X and Y matrices that build the model, respectively, while Q^2^ represents the predictive power of the model. The Q^2^ of each comparison group was greater than 0.78, indicating that the established model was suitable for the analysis of differential metabolites.

In this study, the combined method of Fold Change and VIP was used to screen differential metabolites (FC ≥ 2 or FC ≤ 0.5 and VIP ≥ 1). A total of 141 differential metabolites were identified between the treatment and control groups (CKP1 vs. P1Y2, 92; CKP2 vs. P2Y2, 72; Figure 3C; Supplementary Table S2). Pairwise comparisons were made between the mung bean varieties after Se-enriched treatment to compare the differences between the metabolites of the same mung bean variety. The results showed that there were 92 differential metabolites between CKP1 and P1Y2, of which 53 were upregulated, and 39 were downregulated (Figures 3D,E). These differential metabolites mainly included amino acids and derivatives, phenolic acids, flavonoids, and lipids. There were 72 differential metabolites between CKP2 and P2Y2, of which 30 were upregulated, and 42 were downregulated. These differential metabolites mainly include flavonoids and phenolic acids. Metabolites differed more in P1 than P2 variety, consistent with the HCA and PCA results.

**Figure 4:**
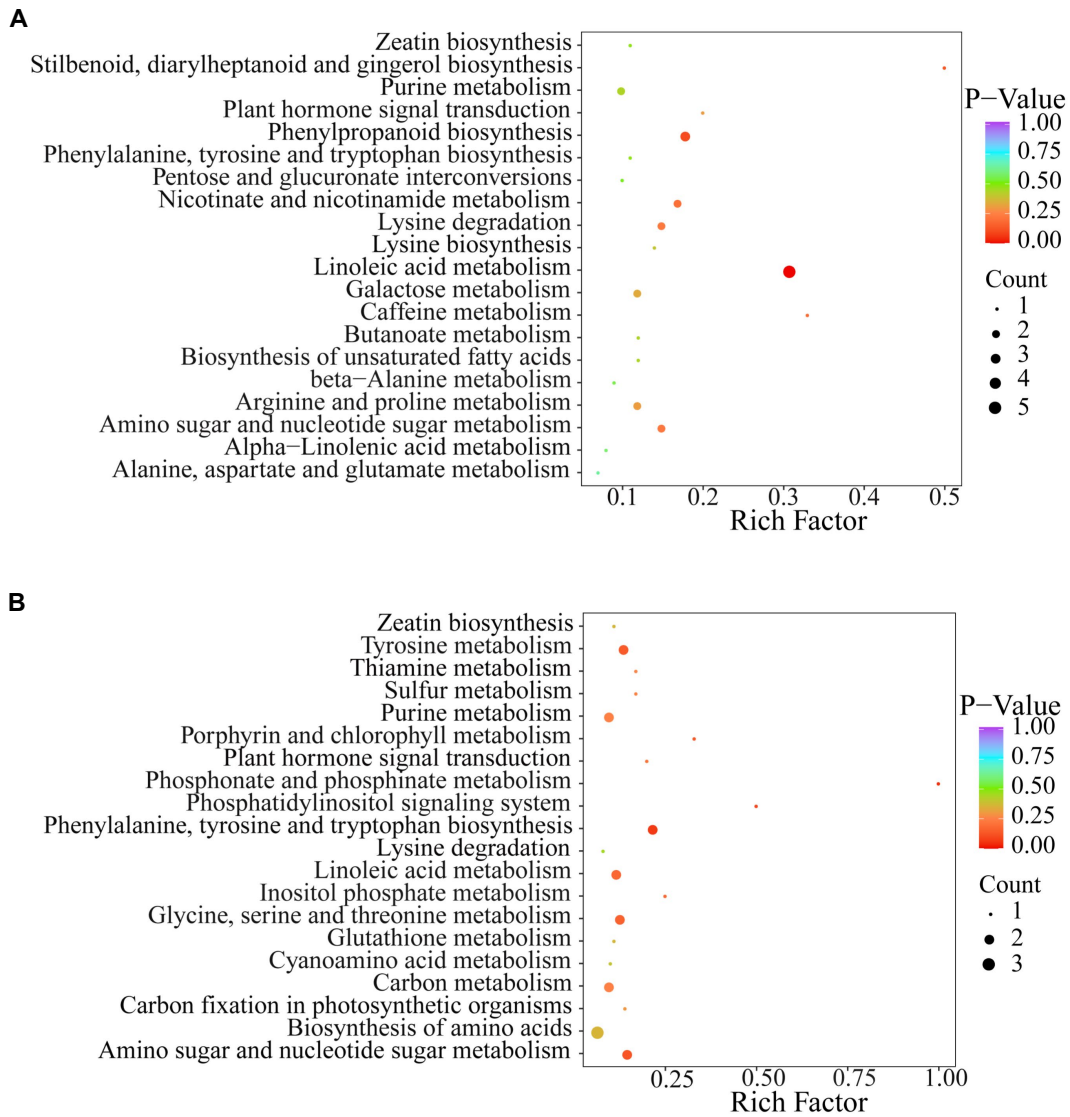
KEGG annotation and biomarker enrichment of black Se-enriched mung bean variety **(A)**, and green Se-enriched mung bean variety **(B)**. Each bubble in the figure represents a metabolic pathway and its abscissa, while the size of the bubble represents the size of the influencing factors of the pathway. Larger bubble size indicates a larger influencing factor. The color of the bubbles represents the value of the p-enrichment analysis, with darker colors indicating higher levels of enrichment.

Kyoto Encyclopedia of Genes and Genomes (KEGG) pathway analysis of differential metabolites was performed to further understand the response of metabolites to exogenous Se application. The results showed that the two Se-enriched mung bean varieties had different metabolic pathways (Figures 4A,B). The main metabolic pathway in the CKP1 vs. P1Y2 treatment group was linoleic acid metabolism. In the CKP2 vs. P2Y2 treatment group, the main metabolic pathways were Phosphonate and phosphinate metabolism. The different metabolites among the Se-enriched mung bean varieties cover many different metabolic pathways, suggesting that the differences may be genotypic dependent.

### Unique metabolites in different varieties of Se-enriched mung bean

Metabolites vary greatly between different varieties of the same plant species. Therefore, a comprehensive comparison of metabolites in different crop varieties can identify specific metabolites that are beneficial to human health (Li et al., 2022). In this study, we screened metabolites that have excellent contributions to the nutritional quality of mung beans (FC ≥ 2 and VIP ≥ 1; Supplementary Table S3). In P1, N-Methyl-L-Glutamic acid and L-Saccharopine were specifically upregulated after Se-enrichment. N-Methyl-L-Glutamate is the downstream metabolite of glutamate, while L-Saccharopine is mainly involved in lysine metabolism. In P2, L-Glycine and S-Allyl-L-cysteine were specifically upregulated after Se-enrichment. S-Allyl-L-cysteine is an intermediate metabolite in the biosynthesis of diallyl thiosulfinate (allicin). It is an organosulfur compound synthesized from allyl mercaptan (Ohsumi et al., 1993; Yamaguchi and Kumagai, 2020).

Regarding lipids, the content of 9S-Hydroxy-10E and 12Z-octadecadienoic acid involved in linoleic acid metabolism increased significantly in P1 after Se-enrichment. In P2, two specifically upregulated metabolites were detected, of which LysoPC 12: 0 was significantly increased after Se-enrichment.

Unique phenolic metabolites were identified in the P1, and all showed significant quantitative differences. Among them, 3-O-Galloyl-D-glucose, a hydrolysate of tannin exhibited the highest accumulation, while the contents of 3-O-Caffeoylquinic acid was were 2090 folds higher than that of the untreated mung bean. In P2, Vnilloylmalic acid was the significantly increased metabolite of phenolic compound metabolite, increasing 13,120 folds higher than that of the CKP2.

Regarding the metabolism of flavonoids, the expression patterns of Tamarixetin-3-O-glucoside and Isovitexin-8-O-xyloside in P1 were highly similar, and their contents increased continuously following Se application. In P2, Isoluteolin-6,8-di-C-glucoside content increased significantly after Se-enrichment, which was 1,714 folds higher than that of the CKP2.

### Identification of biomarkers in Se-enriched mung bean

**Figure 5:**
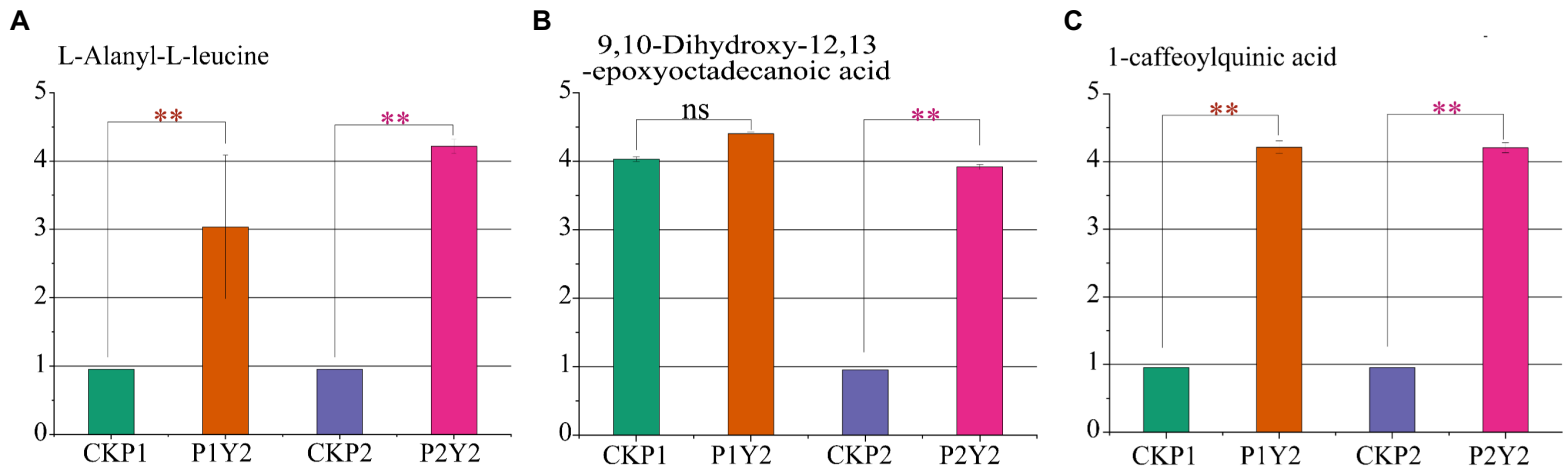
Differences in the relative contents of three nutrient markers in black mung bean variety (P1) and green mung bean variety (P2). L-Alanyl-L-leucine **(A)**, 9,10-Dihydroxy-12,13-epoxyoctadecanoic acid **(B)**, 1-caffeoylquinic acid **(C)**. Logarithmic (log10) transformation of the peak area matrix was performed to reduce the effect of concentration on pattern recognition. “**” means significant (*p* < 0.05), “ns” means not significant.

To identify the differential metabolites shared by Se-enriched mung bean varieties, we made a pairwise comparison of CKP1 vs. P1Y2 and CKP2 vs. P2Y2. A total of 23 overlapping differential metabolites were identified, including 5 amino acids and derivatives, 3 phenolic acids, 2 free fatty acids, 2 flavonols, 2 organic acids, 2 nucleotides and derivatives, 3 others, and 4 triterpene saponins (Supplementary Table S4). To further explore the key metabolites related to the nutritional quality of the Se-enriched mung bean varieties, we took the upregulated metabolites as the standard and screened out three differential metabolites related to nutritional quality in the overlapping substances. Among them, L-Alanyl-L-leucine belongs to amino acids and derivatives, 9,10-Dihydroxy-12,13-epoxyoctadecanoic acid is free fatty acid, 1-caffeoylquinic acid is phenolic acid (Figures 5A–C). These compounds responded specifically to increased nutrient content and thus can serve as biomarkers for selecting Se-enriched mung beans.

## Discussion

In this study, both varieties of mung beans exhibited enhanced levels of Se content and Se-induced nutrient accumulation following exogenous Se application. Different plants have different abilities to transform and enrich Se. Under normal conditions, plant physiological indexes and Se accumulation show nonlinear unimodal response trend, indicating that low-Se dosage can promote crop growth and development, while high dosage can cause crop poisoning (Szarka et al., 2020; Wang et al., 2020). In this study, 30 g ha^−1^ Se concentration significantly increased the contents of protein, fat, total phenols, and total flavonoids in the two mung bean varieties. However, the protein and total flavonoid contents in P1 and the total phenolic content in P2 decreased gradually at 45 g ha^−1^ Se. Therefore, Se-enriched mung beans also showed a nonlinear unimodal response trend between nutrients and exogenous Se concentration. Nevertheless, the protein, total flavonoids, and total phenolic contents were still higher than that of the control group at 45 g ha^−1^ Se concentration, indicating that the maximum Se concentration used in this study did not exceed the toxicity threshold. These results also show that mung bean has a high tolerance to Na_2_SeO_3_. The bioavailability of Fe, Zn, Cu, and Mg in human diets is often reduced by the presence of phytic acid, which is considered an antinutrient (Raboy, 2003). We found that Se fertilization did not alter phytic acid content. Silva et al. (2019) also confirmed this result. Therefore, Se biofortification of mung bean does not negatively affect the bioavailability of the nutrient in humans. Gąsecka et al. (2015) demonstrated that Se fertilizer increases the total content of phenols and flavonoids in plants. Liang et al. (2020) found that the application of Se can down-regulate the synthesis of phospholipase, and phospholipase is involved in lipid degradation, so the down-regulation of phospholipase is beneficial to lipid synthesis. Also, Se fertilization has been shown to promote the metabolism of serine, glycine, threonine, and β-alanine, leading to an increase in the content of total protein in plants (Liang et al., 2020). Currently, metabolic profiling has been successfully applied to distinguish different plant phenotypes and provide potential biomarkers for controlling food quality (Sumner et al., 2015). In the present study, we employed metabolomics to further explore the mechanism of Se fertilization in improving the nutritional quality of mung beans. A total of 1,080 metabolites were identified, of which the metabolites related to the synthesis of protein, fat, total phenols, and total flavonoids accounted for 63.24% of the total metabolites. Screening of metabolites with positive regulatory effects revealed that L-Alanyl-L-leucine, 9,10-Dihydroxy-12,13-epoxyoctadecanoic acid, and 1-caffeoylquinic acid were the key metabolites in Se-enriched mung bean.

In the present study, we analyzed the amino acids and derivatives, lipids, phenolic acids, and flavonoid metabolites in different varieties of mung beans to explore the reasons that affect their nutritional quality. Amino acids are the core units of proteins that give them their specific molecular structure. The composition and abundance of amino acids are key indicators of the nutritional quality of grains. We found that Se-enriched treatment could promote the synthesis of glutamate and lysine in mung bean. Luo et al. (2021) confirmed that exogenous Se application increases glutamate synthesis. Lysine is a limiting amino acid and one of the essential amino acids necessary for the normal growth and development of the human. However, human and animal bodies cannot synthesize lysine (Liu et al., 2019). Therefore, mung bean is of great significance for the synthesis of lysine after Se application. Notably, the increase in the content of S-Allyl-L-cysteine, a sulfur-containing metabolite, indicates that changes in plant utilization of Se are associated with changes in sulfur uptake. Sulfur and Se are chemically similar and share a common metabolic pathway, thus they can be transported and assimilated into similar amino acids by the same membrane transporters, resulting in changes in sulfur-containing secondary metabolites (Sors et al., 2005; Malagoli et al., 2015). S-Allyl-L-cysteine exerts powerful antioxidant properties by scavenging superoxide radicals, hydroxyl radicals, hydrogen peroxide, and lipid peroxidation (Ide and Lau, 2001; Numagami and Ohnishi, 2001). It also regulates NO production to play an anti-inflammatory role (Kim et al., 2001; Cheng et al., 2021). Furthermore, L-Glycine can act on inflammatory cells, such as macrophages, to inhibit the activation of transcription factors and the formation of free radicals and inflammatory cytokines (Zhong et al., 2003). Therefore, Se-induced upregulation of amino acids and derivatives increased protein content and enhanced antioxidant capacity by affecting various physiological mechanisms. Lipids and their components are essential for participating in cell metabolism and maintaining the fluidity and integrity of cell membranes. In the present study, 9 s-hydroxy-10e and 12z-octadecadienoic acid promoted the synthesis of linoleic acid. Linoleic acid is the most abundant polyunsaturated fatty acid in human low-density lipoprotein and is an essential nutrient for the human body (Wang et al., 2009). Interestingly, Se fertilization promotes upregulation of LysoPC 12: 0. As an isomer of lysophosphatidylcholine, LysoPC 12: 0 is an important part of the cell membrane and participates in clathrin-mediated endocytosis, phagocytosis, pinocytosis, and other physiological and biochemical functions. LysoPC 12: 0 is also a nutrient that is widely present in food crops and has significant physiological effects on the biotic and abiotic stress responses in plants (Law et al., 2019). Generally, the active antioxidant components of plants contribute significantly to their nutritional value. Exogenous application of Se can enhance the accumulation of anthocyanins, total phenols, total flavonoids, carotenoids, and ascorbic acid in fruits, vegetables, and grain products (Schiavon et al., 2016; D Amato et al., 2020; Islam et al., 2020; Sun et al., 2021). Polyphenols are antioxidant substances. Phenolic acids and flavonoids are important components of polyphenols and are closely associated with the antioxidant activity of the plant. We analysis of phenolic acids of the Se-enriched mung beans revealed that 3-O-Caffeoylquinic acid accounted for a large proportion of the metabolites. Caffeoylquinic acid derivatives have been shown to have a strong positive correlation with total phenolic content, suggesting that the increase in total phenolic content in Se-enriched mung beans may be related to the upregulation of caffeoylquinic acid derivatives (Islam et al., 2003). Synthesis of phenolic substances can inhibit DPPH and ABTS free radicals, increase antioxidant activity, and have a certain scavenging effect on peroxynitrite-induced inflammation (Suschek et al., 2004; Xu et al., 2021). Flavonoids are secondary metabolites widely present in higher plants and can protect the human body from oxidative stress. The variations in phenolic acid and flavonoid of the two varieties of mung beans following Se application indicate that exogenous Se potentially enhances the content of total phenols and total flavonoids in plants, thereby improving their antioxidant properties. Based on the present results, this study showed that 30 g ha^−1^ of Se had a promoting effect on the improvement of mung bean nutritional quality (protein, fat, total phenols, and total flavonoids). Further analysis using metabolomics technology found that exogenous Se had an effect on the accumulation of primary metabolites (amino acids and their derivatives and lipids) and secondary metabolites (phenolic acids and flavonoids), such as significantly promoted the synthesis of N-Methyl-L-Glutamate, L-Saccharopine, S-Allyl-L-cysteine, 9S-Hydroxy-10E,12Z-octadecadienoic acid, LysoPC 12: 0. This not only contributes to the synthesis of protein and fat but also enhances the resistance of mung bean to biotic and abiotic stresses. Secondary metabolites may play an important role as antioxidants and regulatory substances, 1-caffeoylquinic acid, 3-O-Galloy-D-glucose, 3-O-caffeoylquinic acid, and other secondary metabolites are significantly affected by Se, which has a certain effect on increasing the contents of total phenols and flavonoids. In addition, these metabolites have special nutritional and medicinal value, and also greatly increase the application value of Se-enriched mung beans. Overall, mung beans are excellent target for Se biofortification, which can significantly affect the nutrition and antioxidant capacity of plants, and people can supplement this essential micronutrient by consuming Se-enriched mung beans.

## Data availability statement

The datasets presented in this study can be found in online repositories. The names of the repository/repositories and accession number(s) can be found in the article/Supplementary material.

## Author contributions

KW designed the research. XL and ZS participated in the conceptualization and contributed to software. YH and HZ performed the funding acquisition and data analysis. XG discussed the results and wrote the manuscript. All authors contributed to the article and approved the submitted version.

## Funding

The authors are grateful for the financial support of the National Key Research and Development Program of China (nos. 2021YFD1600600 and 2021YFD1600605).

## Conflict of interest

The authors declare that the research was conducted in the absence of any commercial or financial relationships that could be construed as a potential conflict of interest.

## Publisher’s note

All claims expressed in this article are solely those of the authors and do not necessarily represent those of their affiliated organizations, or those of the publisher, the editors and the reviewers. Any product that may be evaluated in this article, or claim that may be made by its manufacturer, is not guaranteed or endorsed by the publisher.
