# Supplementary material for: Effects of exogenous selenium application on nutritional quality and metabolomic characteristics of mung bean (Vigna radiata L.)
Source: Front Plant Sci. 2022 Aug 18;13:961447. doi: 10.3389/fpls.2022.961447 (PMC9433778; doi:10.3389/fpls.2022.961447)
Supplement: Supplementary file 1 [file Data_Sheet_1.ZIP › supplementary date/Supplementary data 3.docx]

Unique differential metabolites in black mung bean

| **_Class_** | **_Compounds_** | **_Peak area_** | | **_P_** | **_Fold-Chang_** | **_VIP(＞1)_** |
| --- | --- | --- | --- | --- | --- | --- |
|  |  | **_CKP1_** | **_P1Y2_** |  |  |  |
| Amino acids and derivatives | N-Methyl-L-Glutamate | 8683±374 | 19233±4318 | 0.13 | 2.22 | 1.26 |
|  | L-Saccharopine | 22233±3372 | 52033±7817 | 0.05 | 2.34 | 1.33 |
| Phenolic acids | Ethylparaben | 9343±475 | 18800±5651 | 0.24 | 2.01 | 1.10 |
|  | MethylEugenol | 9±0 | 13633±1105 | 0.01 | 1520.00 | 1.51 |
|  | Sinapic acid | 9±0 | 4147±1116 | 0.07 | 461.00 | 1.51 |
|  | 3-O-Galloyl-D-glucose* | 74333±11177 | 185000±14978 | 0.01 | 2.49 | 1.41 |
|  | 3-O-Caffeoylquinic acid* | 9±0 | 18833±4278 | 0.05 | 2090.00 | 1.51 |
|  | 3-Prenyl-4-O-glucosyloxy-4-hydroxybenzoic Acid | 9±0 | 1008±282 | 0.07 | 112.00 | 1.50 |
|  | Dicaffeoylshikimic acid | 9±0 | 2236±1114 | 0.18 | 249.00 | 1.07 |
| Flavonoids | Quercetin-3-O-xyloside (Reynoutrin) | 9±0 | 10170±2888 | 0.07 | 1130.00 | 1.51 |
|  | Epicatechin gallate* | 2797± 348 | 7340± 614 | 0.01 | 2.63 | 1.44 |
|  | Tamarixetin-3-O-glucoside (Tamarixin) | 9±0 | 25067±713 | 0.00 | 2790.00 | 1.51 |
|  | Syringetin-7-O-glucoside | 6297±1275 | 15533± 633 | 0.01 | 2.47 | 1.37 |
|  | Isovitexin-8-O-xyloside | 55767± 240 | 157000±2082 | 0.00 | 2.81 | 1.51 |
|  | Apigenin-8-C-(2''-xylosyl)glucoside | 3003± 8762 | 662000± 38158 | 0.01 | 2.20 | 1.49 |
|  | Catechin-catechin-catechin | 9± 0 | 6293± 1334 | 0.04 | 699.00 | 1.51 |
| Lipids | 13-methylmyristic acid | 5839±5830 | 17133± 1013 | 0.19 | 2.93 | 1.07 |
|  | 17-Hydroxylinolenic acid | 23233±560 | 73800± 3219 | 0.00 | 3.17 | 1.51 |
|  | 13(S)-HODE;13(S)-Hydroxyoctadeca-9Z,11E-dienoic acid | 14033±1201 | 288333± 20610 | 0.02 | 2.05 | 1.48 |
|  | 9S-Hydroxy-10E,12Z-octadecadienoic acid | 16100± 1732 | 336667± 16676 | 0.01 | 2.09 | 1.50 |
|  | Eicosenoic acid | 244333± 64191 | 67666± 148723 | 0.08 | 2.77 | 1.22 |
|  | Hydroxy ricinoleic acid | 113667± 4841 | 261000± 10817 | 0.00 | 2.30 | 1.50 |
|  | 9,10,13-Trihydroxy-11-Octadecenoic Acid | 90667± 6562 | 211333 ±7265 | 0.00 | 2.33 | 1.49 |
|  | 1-(9Z-Octadecenoyl)-2-(9-oxo-nonanoyl)-sn-glycero-3-phosphocholine | 9± 0 | 6233± 1279 | 0.04 | 692.00 | 1.51 |

Data are in the form of mean ± SE of three replications

| **Class** | **Compounds** | **Peak area** | | **P** | **Fold-Chang** | **VIP(＞1)** |
| --- | --- | --- | --- | --- | --- | --- |
|  |  | **CKP2** | **P2Y2** |  |  |  |
| Amino acids and derivatives | L-Glycine | 9±0 | 14944±5172 | 0.10 | 1660.43 | 1.76 |
|  | S-Allyl-L-cysteine | 9±0 | 37280±751 | 0.00 | 4142.19 | 1.78 |
| Phenolic acids | Tyrosol | 9±0 | 15967±3270 | 0.04 | 1774.14 | 1.77 |
|  | Methyl gallate | 9±0 | 13408±1378 | 0.01 | 1489.78 | 1.77 |
|  | Ferulic acid methyl ester | 9±0 | 7741±1102 | 0.02 | 860.10 | 1.77 |
|  | Vnilloylmalic acid | 9±0 | 118077±8544 | 0.01 | 13119.63 | 1.77 |
|  | Vnilloyltartaric acid | 9±0 | 7206±512 | 0.01 | 800.65 | 1.77 |
|  | 2-O-Galloyl-D-glucose | 9±0 | 27831±3877 | 0.02 | 3092.37 | 1.77 |
| Flavonoids | Chrysin | 9±0 | 3681±254 | 0.00 | 408.95 | 1.77 |
|  | 6-HydroxyLuteolin | 9±0 | 3060±140 | 0.00 | 339.95 | 1.77 |
|  | Phloretin-4'-O-(6''-p-Coumaroyl)glucoside | 9±0 | 1528±198 | 0.02 | 169.78 | 1.77 |
|  | Luteolin-7-O-gentiobioside | 9±0 | 7266±1457 | 0.04 | 807.37 | 1.77 |
|  | Isoluteolin-6,8-di-C-glucoside | 9±0 | 15428±945 | 0.00 | 1714.22 | 1.77 |
|  | Quercetin-3-O-(6''-O-galloyl)galactoside | 9±0 | 1380±384 | 0.07 | 153.38 | 1.76 |
|  | Kaempferol-3-O-glucuronide-7-O-glucoside | 9± 0 | 2662±1195 | 0.16 | 295.82 | 1.75 |
|  | Isorhamnetin-3-O-glucoside-7-O-rhamnoside | 9±0 | 7745±1479 | 0.03 | 860.59 | 1.77 |
|  | Isorhamnetin-3-O-sophoroside | 9±0 | 4701±552 | 0.01 | 522.32 | 1.77 |
| Lipids | Hexadecylsphingosine | 1618020± 931779 | 3622067± 147460 | 0.16 | 2.24 | 1.27 |
|  | LysoPC 12:0 | 9±0 | 3141±405 | 0.02 | 348.97 | 1.77 |

Unique differential metabolites in mung bean

Data are in the form of mean ± SE of three replications
